# Association of dynamic contrast-enhanced MRI and ^18^F-Fluorodeoxyglucose PET/CT parameters with neoadjuvant therapy response and survival in esophagogastric cancer

**DOI:** 10.1016/j.ejso.2023.05.009

**Published:** 2023-10

**Authors:** Samuel J. Withey, Kasia Owczarczyk, Mariusz T. Grzeda, Connie Yip, Harriet Deere, Mike Green, Nick Maisey, Andrew R. Davies, Gary J. Cook, Vicky Goh, Guy's & St Thomas' Esophagogastric Research Group, C.R. Baker, J. Bell, F. Chang, S. Chicklore, M. Cominos, A. Coombes, J.N. Dunn, S. George, B. Gill-Barman, J.A. Gossage, S. Gourtsoyianni, A. Green, N. Griffin, M. Hill, O. Hynes, C. Iezzi, A. Jacques, M. Kelly, U. Mahadeva, R. McEwan, J. Meenan, R. Neji, S. Ngan, F. Padormo, A. Qureshi, A. Reyhani, A.R. Sharkey, J. Spence, M. Subesinghe, G. Tham, J. Waters, S.S. Zeki

**Affiliations:** a Department of Radiology, Guy's and St Thomas' Hospitals NHS Foundation Trust, London, United Kingdom; b Department of Clinical Oncology, Guy's and St Thomas' Hospitals NHS Foundation Trust, London, United Kingdom; c School of Biomedical Engineering & Imaging Sciences, King's College London, United Kingdom; d Department of Radiation Oncology, National Cancer Centre, Singapore; e Department of Pathology, Guy's and St Thomas' Hospitals NHS Foundation Trust, London, United Kingdom; f Department of Medical Oncology, Guy's and St Thomas' Hospitals NHS Foundation Trust, London, United Kingdom; g Department of Surgery, Guy's and St Thomas' Hospitals NHS Foundation Trust, London, United Kingdom; h The King's College London and Guy's and St Thomas' PET Centre, St Thomas' Hospital, London, United Kingdom

**Keywords:** Magnetic resonance imaging, Perfusion imaging, Positron emission tomography computed tomography, Fluorodexoyglucose F18, Esophageal cancer

## Abstract

**Introduction:**

Better predictive markers are needed to deliver individualized care for patients with primary esophagogastric cancer. This exploratory study aimed to assess whether pre-treatment imaging parameters from dynamic contrast-enhanced MRI and ^18^F-fluorodeoxyglucose (^18^F-FDG) PET/CT are associated with response to neoadjuvant therapy or outcome.

**Materials and methods:**

Following ethical approval and informed consent, prospective participants underwent dynamic contrast-enhanced MRI and ^18^F-FDG PET/CT prior to neoadjuvant chemotherapy/chemoradiotherapy ± surgery. Vascular dynamic contrast-enhanced MRI and metabolic ^18^F-FDG PET parameters were compared by tumor characteristics using Mann Whitney *U* test and with pathological response (Mandard tumor regression grade), recurrence-free and overall survival using logistic regression modelling, adjusting for predefined clinical variables.

**Results:**

39 of 47 recruited participants (30 males; median age 65 years, IQR: 54, 72 years) were included in the final analysis. The tumor vascular-metabolic ratio was higher in patients remaining node positive following neoadjuvant therapy (median tumor peak enhancement/SUV_max_ ratio: 0.052 vs. 0.023, p = 0.02). In multivariable analysis adjusted for age, gender, pre-treatment tumor and nodal stage, peak enhancement (highest gadolinium concentration value prior to contrast washout) was associated with pathological tumor regression grade. The odds of response decreased by 5% for each 0.01 unit increase (OR 0.95; 95% CI: 0.90, 1.00, p = 0.04). No ^18^F-FDG PET/CT parameters were predictive of pathological tumor response. No relationships between pre-treatment imaging and survival were identified.

**Conclusion:**

Pre-treatment esophagogastric tumor vascular and metabolic parameters may provide additional information in assessing response to neoadjuvant therapy.

## 1 Introduction

Esophageal cancer, including cancers extending to the esophagogastric junction, affects 456,000 new patients worldwide each year [1]. It is a leading cause of cancer death with 5-year overall survival rates of 15%–25%. For suitable patients presenting with localized disease, clinical guidelines recommend neoadjuvant chemotherapy or chemoradiotherapy followed by definitive surgery [2]. Nevertheless, only 30–40% of patients achieve a ‘cure’ [3], but all experience a significant negative impact on quality of life [4]. Better patient stratification for neoadjuvant treatment remains a key challenge [5]. Reliable pre-treatment predictive markers could potentially allow for intensification of neoadjuvant treatment in resistant tumor phenotypes, or omission of surgery in complete responders, and improve patient reported outcome measures. The aim of this prospective exploratory study was to determine whether tumor vascular and metabolic parameters derived from dynamic contrast-enhanced MRI and ^18^F-fluorodeoxyglucose (^18^F-FDG) PET/CT, respectively, may provide additional predictive and/or prognostic information to current staging.

## 2 Materials and methods

### 2.1 Participants

**Fig. 1:**
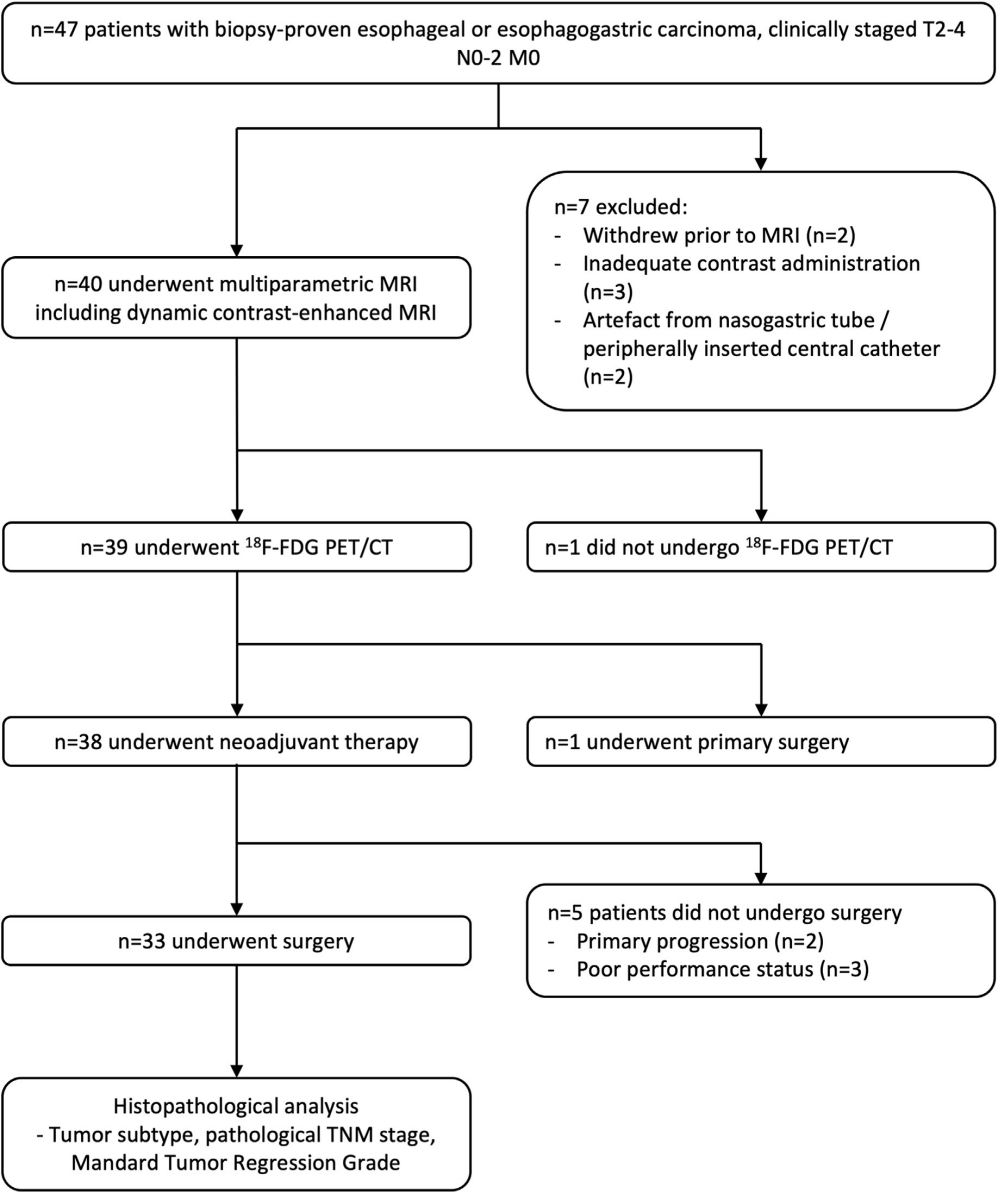
Participant flow through the study.

Following ethical approval and written informed consent, consecutive participants with a new diagnosis of thoracic esophageal/esophagogastric cancer, and under consideration for definitive treatment, were recruited between March 2014 and March 2020, when recruitment was suspended due to the COVID pandemic. Inclusion criteria were adults with histologically proven cancer; Stage II-III (T2-4, N0-3, M0; American Joint Committee on Cancer TNM (tumor, node, metastasis) staging system, 7th edition [6]); ECOG performance status 0–2; who were candidates for definitive treatment (surgery ± neoadjuvant chemotherapy or chemoradiation; or definitive chemoradiation). Pre-treatment staging was the tumor board clinical TNM stage. For nodal status, this took into account size criteria from CT (short axis >10 mm), metabolic activity on 18 F-FDG PET/CT, and endoscopic ultrasound±fine needle aspiration unless the tumor was non-passable at endoscopy. Exclusion criteria included inability to consent; presence of distant metastatic disease; any contraindication to MRI contrast agent administration; prior mucosal resection of the tumor; and prior thoracic radiotherapy or systemic chemotherapy, within the preceding 3 months. Fig. 1 summarizes the participant flowchart.

### 2.2 Imaging

#### 2.2.1 MRI

1.5 T MRI (Magnetom Aera, Siemens Healthcare) was performed using an 18-channel body and a 32-channel spine coil, centered on the primary tumor, prior to commencement of neoadjuvant chemotherapy or chemoradiation, and within a mean ± SD of 19 ± 9 days of the staging ^18^F-FDG PET/CT. Hyoscine butylbromide (Buscopan, Boehringer Ingelheim) 20 mg was administered intravenously as an anti-peristaltic, unless contraindicated. In addition to standard diagnostic sequences, dynamic T1-weighted gradient echo sequences to assess tumor vascularization were acquired in the axial plane following administration of gadolinium-based contrast agent (gadoterate meglumine, Dotarem, Guerbet) 0.2 ml/kg, injected using a pump at 4 ml/s, followed by a 20 ml saline chaser. MRI acquisition parameters are summarized in Supplemental Table 1.

#### 2.2.2 ^18^F-FDG PET/CT

^18^F-FDG PET/CT (Discovery 710, GE Healthcare) was performed after intravenous injection of up to 400MBq ^18^F-FDG and an uptake period of 60 min, provided the pre-imaging blood glucose level was ≤10 mmol/L. Imaging extended from the skull base to mid-thigh. PET scan duration was 3 min per bed position. PET image reconstruction included standard scanner-based corrections for radiotracer decay, scatter, randoms and dead-time. Emission sinograms were reconstructed with a time-of-flight ordered subset expectation maximization algorithm and Gaussian post-reconstruction smoothing filter. A low dose CT scan was performed at the start of imaging to provide attenuation correction and an anatomical reference standard. ^18^F-FDG PET/CT acquisition parameters are summarized in Supplemental Table 2.

### 2.3 Image analysis

#### 2.3.1 MRI

Dynamic contrast-enhanced MRI was analyzed using Tissue 4D (Syngo.via, Siemens Healthcare) allowing both qualitative and quantitative assessment (pharmacokinetic modelling). Following motion correction and image registration of the pre-contrast and dynamic contrast-enhanced T1-weighted sequence, the tumor was located by two radiologists in consensus. A volume-of-interest encompassed the tumor craniocaudally (but excluding the most cranial or caudal slices to minimize inflow-outflow effects), generating averaged parameter values for each patient.

The following qualitative parameters were recorded for the primary tumor.

• Initial area under the gadolinium concentration-time curve (iAUGC); within 60 s of the calculated contrast arrival time, reflecting inflow and vascular leakage
• Peak enhancement; highest value of Gadolinium concentration, prior to washout
• Time to peak enhancement (TTP, min)

Following pharmacokinetic modelling using the Toft's model [7] with the computationally-efficient population-based vascular input described by Orton [8], the following quantitative parameters were recorded.

• Transfer constant (K^trans^, min^−1^); rate of leakage of gadolinium from blood plasma to the extracellular extravascular space (EES)
• Relative volume of EES (V_e_, range 0–1); relative amount of interstitial space available to accumulate gadolinium
• Rate constant (k_ep_, min^−1^); rate of reflux of gadolinium from the EES, back into the vasculature

#### 2.3.2 ^18^F-FDG PET/CT

^18^F-FDG PET/CT analysis with a pre-defined threshold of 40% of the maximum voxel intensity was performed by a single nuclear medicine/PET physician with >20 years of experience, blinded to MRI and clinical data), by placing a bounding box around the tumor using a standard clinical platform (HybridViewer, Hermes Medical Solutions). Maximum and mean standardized uptake values (SUV_max_ and SUV_mean_, respectively), metabolic tumor volume (MTV) and total lesion glycolysis (TLG = SUV_mean_ x MTV) were recorded.

### 2.4 Treatment

#### 2.4.1 Neoadjuvant treatment

Patients were treated with either neoadjuvant chemoradiation as per the CROSS protocol [9], receiving 41.4Gy in 23 fractions over five weeks with concomitant weekly carboplatin AUC 2 and paclitaxel 50 mg/m^2^ or with neoadjuvant ECX chemotherapy (epirubicin 50 mg/m^2^ on day 1, cisplatin 60 mg/m^2^ on day 1 and capecitabine 625 mg/m^2^ bd day 1–21; every 21 days for 3 cycles) or neoadjuvant FLOT chemotherapy (docetaxel 50 mg/m^2^ day 1, oxaliplatin 85 mg/m^2^ day 1 and 5-fluorouracil 2600 mg/m^2^ administered as a continuous infusion over 24 h every 14 days for 4 cycles) [10].

#### 2.4.2 Definitive treatment

Patients who were deemed fit for surgery underwent a *trans*-hiatal or *trans*-thoracic esophagectomy with en-bloc lymphadenectomy [11]. Patients who were not surgical candidates, underwent definitive chemoradiation to a dose of 50Gy in 25 fractions over 5 weeks with concomitant cisplatin 60 mg/m^2^ and either 5-fluorouracil administered as a continuous infusion 1000 mg/m^2^ on day 1–4 or capecitabine 625 mg/m^2^ twice daily on day 1–25 for 4 cycles [12]. Radiotherapy was planned using a 3- or 4-dimensional CT simulation. Clinical target volume was defined as the gross tumour volume (contoured in reference to the imaging and endoscopy report) expanded by 2 cm superiorly and inferiorly along the body of the esophagus (or 1 cm above the most superior or inferior involved node) and 1 cm laterally, anteriorly and posteriorly edited for lung, pericardium and vertebral body. Clinical target volume was expanded using a uniform 0.5 cm margin to form the planning treatment volume [13]. Radiotherapy was delivered using intensity modulated radiotherapy (IMRT) or volumetric arc radiotherapy (VMAT) technique.

### 2.5 Histopathological assessment

Pathologic tumor regression following neoadjuvant treatment was assessed as standard by 2 gastrointestinal pathologists using the Mandard tumor regression grade (TRG), where TRG 1 represents complete regression (no viable tumor cells); TRG 2 represents fibrosis with rare tumor cells; TRG 3 represents fibrosis and tumor cells, with preponderance of fibrosis; TRG 4 represents fibrosis and tumor cells, with a preponderance of tumor cells; and TRG 5 represents tumor without evidence of regression [14]. Post-treatment nodal status was determined by assessment of all resected nodes for presence/absence of viable tumour cells.

### 2.6 Follow-up

Patients were reviewed every 3 months for the first 2 years and every 6 months thereafter with yearly CT scan as per institutional practice for up to 5-years. Increased frequency of imaging and use of endoscopy were dependent on patient symptoms. An event constituted recurrence (local or metastatic) or death. Follow-up was censored at the last clinic appointment or date of the last surveillance scan.

### 2.7 Statistical analysis

#### 2.7.1 Association between imaging parameters and tumor characteristics

Analysis was undertaken by a statistician using Stata, version 15.1 (StataCorp LP). Pearson correlation coefficient assessed the relationship between dynamic contrast-enhanced MRI and PET parameters. Mann-Whitney *U* test compared the tumor vascular-metabolic ratio (peak enhancement/SUV_max_ or K^trans^/SUV_max_) by pathological tumor stage (T1/2 versus T3/4), nodal status (negative versus positive), and resection margin status (negative versus positive).

#### 2.7.2 Associations with outcomes

Associations between imaging variables obtained from dynamic contrast-enhanced MRI and ^18^F-FDG PET/CT and outcome (response or survival) variables were assessed. The Mandard TRG score was included in the analyses as a binary variable with ‘1’ corresponding to TRG 1–2 (good response) and ‘0’ corresponding to TRG 3–5 (poor response) [15]. Recurrence-free and overall survival time was represented in the analyses as months elapsed from treatment to an event or censoring. All statistical hypotheses were tested at alpha = .05 (type I error), taking into consideration the limited sample size and exploratory nature of the analysis in drawing conclusions about investigated associations.

To assess associations with response, descriptive statistics summarizing distributions of tumor characteristics across groups determined by values on Mandard TRG scores were calculated initially. Since the majority of imaging derived variables were non-normally distributed medians and interquartile ranges (IQR) were reported along with results of Mann-Whitney U tests. All imaging variables were transformed into their z-scores (having 1SD as a unit, where the variable value was subtracted from the mean and divided by the SD) prior to univariate and multivariable logistic regression modelling for the outcome of interest. Effects were reported by odds ratio (OR), 95% confidence intervals and p-values and for multivariable modelling, effect was adjusted by pre-specified baseline clinical information - age, gender, T stage and N stage. The goodness of fit of each model was summarized with the area under the receiver operating characteristic curve (AUC). To assess associations with survival, again standardized forms (z-scores) were entered individually to a series of univariate Cox regression models. Results of these analyses were reported as hazard ratios (HR) along with their 95% confidence intervals and p-values.

## 3 Results

### 3.1 Participant and tumor characteristics

47 participants were recruited. 39 participants (30 male, 9 female; median (IQR) age 65 (54–72) years) completed baseline staging ^18^F-FDG PET/CT and dynamic contrast-enhanced MRI and included in this analysis (Fig. 1). The majority of participants had ≥ T3 stage (88%; 35/39), node positive tumors (79%; 29/39). 85% (33/39) of tumors were adenocarcinomas. 95% (37/39) were located at the esophagogastric junction or lower esophagus. Surgery was performed following neoadjuvant therapy in 85% (33/39). The majority of patients received neoadjuvant FLOT chemotherapy prior to surgery (91%, 30/33). 33% (11/33) of the patients who underwent surgery demonstrated a good pathological response (Mandard TRG 1 or 2). Table 1 summarizes participant and tumor characteristics. No correlations were identified between dynamic contrast-enhanced MRI and ^18^F-FDG PET/CT variables (r: −0.32 to 0.15; p > 0.05). There was a difference in vascular-metabolic ratio (peak enhancement: SUV_max_) in node positive versus node negative participants (median, 19.3 vs. 44.1, p = 0.02) but not for higher T-stage (median, 0.046 vs. 0.044, p = 0.62) or resection margin positivity (median, 0.039 vs. 0.045, p = 0.73).

**Table 1:** Participant & tumor characteristics (n = 39).

	Number (n)	Percentage (%)
Gender		
Female	9	23
Male	30	77
**Age**		
<60 years	15	38
≥60 years	24	62
**T stage**		
T2	4	10
T3	34	87
T4	1	3
**N stage**		
N0	8	21
N1	16	41
N2	13	33
N3	2	5
**Histology**		
Adenocarcinoma	33	85
Squamous cell carcinom	6	15
**Tumor location**		
Mid thoracic esophagus	2	5
Low thoracic esophagus	17	44
Esophagogastric	20	51
**Treatment**		
Surgery alone	1	3
Chemoradiation alone	3	8
∗Chemotherapy alone	2	5
aNeoadjuvant	3	8
chemoradiation +		
surgery		
bNeoadjuvant	30	77
hemotherapy + surgery		
**Surgical resection margins**		
R0	32	94
R1	2	6

∗ ECX chemotherapy (epirubicin, cisplatin, capecitabine);

a Carboplatin, paclitaxel plus radiotherapy.

b FLOT chemotherapy (docetaxel, oxaliplatin, 5-fluorouracil).

### 3.2 Associations with neoadjuvant therapy response

**Fig. 2:**
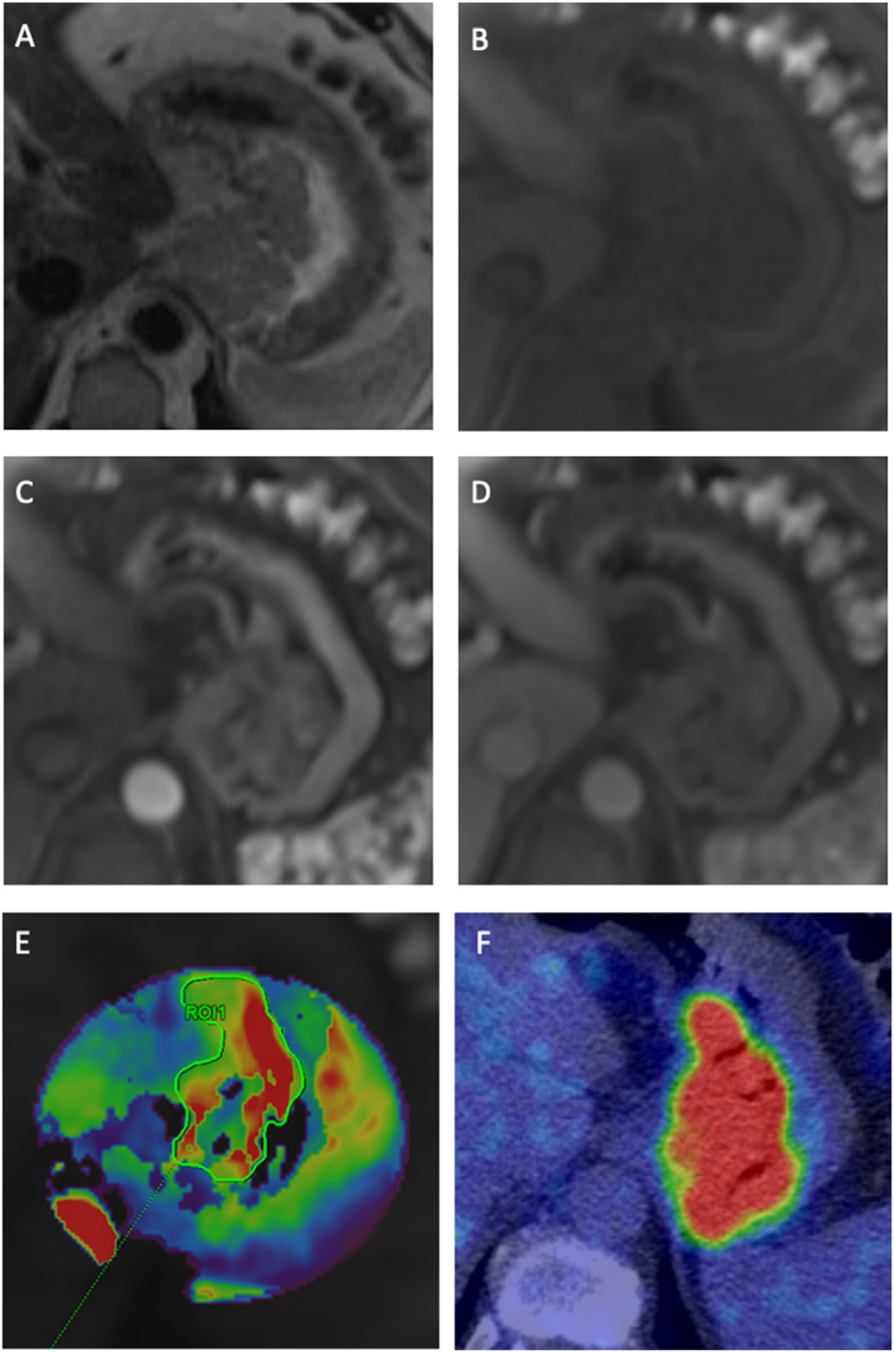
59-year-old with an esophagogastric adenocarcinoma responding to neoadjuvant therapy (TRG 2). Axial T2-weighted MRI (A) demonstrates the primary tumor. Axial pre-contrast T1-weighted MRI (B), arterial phase T1-weighted MRI (C) and portal-venous phase T1-weighted MRI (D) show early tumor enhancement with washout. Corresponding peak enhancement map (E) and ^18^F-FDG PET/CT (F) show lower tumor peak enhancement of 0.17 mmol.L^−1^ and metabolic activity, SUV_max_ of 29.8, respectively.

**Fig. 3:**
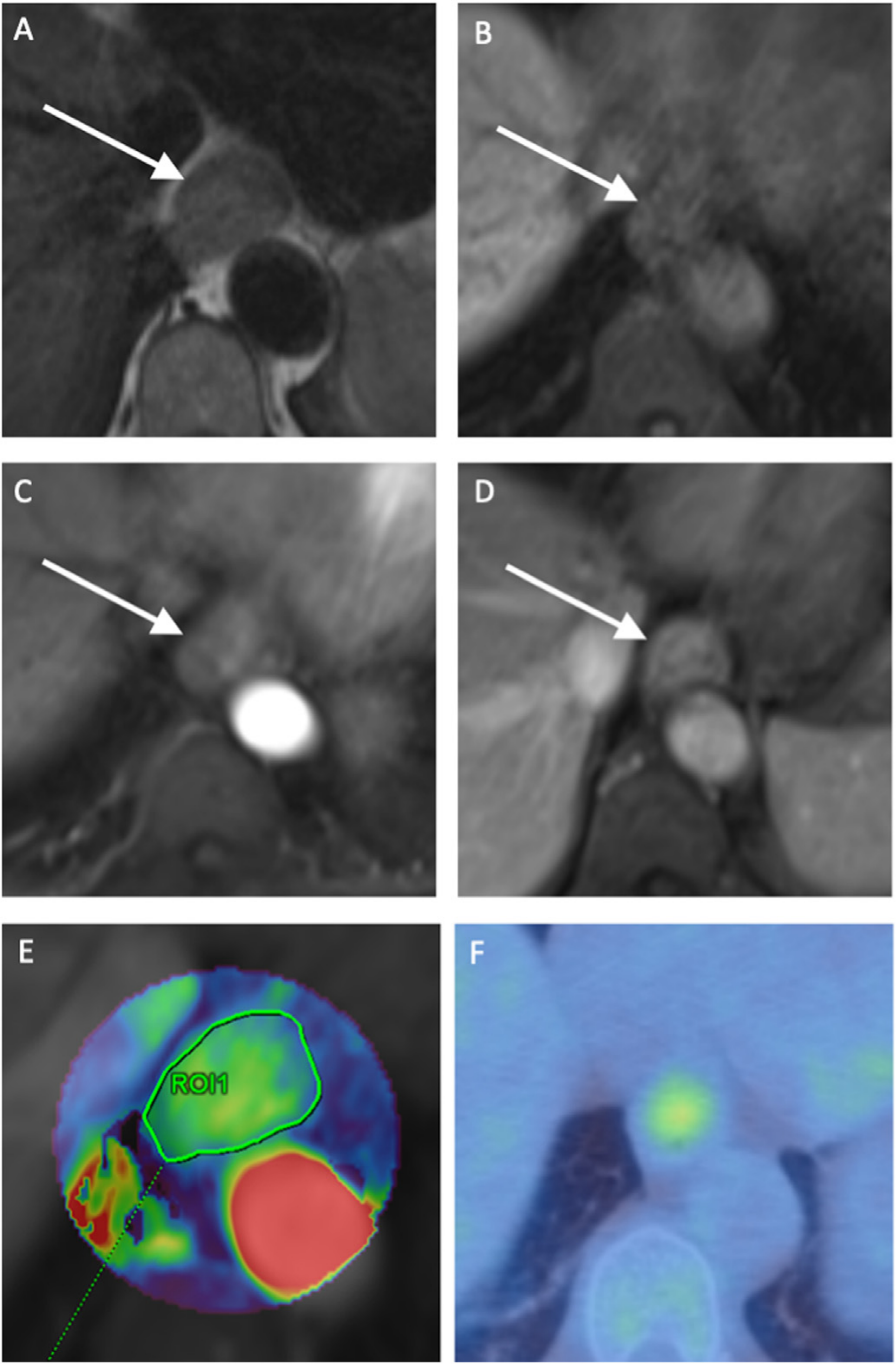
75-year-old with a lower esophageal adenocarcinoma who had a poor response to neoadjuvant therapy (TRG 4). Axial T2-weighted MRI image (A) demonstrates the primary tumor. Axial pre-contrast T1-weighted MRI image (B), axial arterial phase T1-weighted MRI image (C) and axial portal-venous phase T1-weighted MRI image (D) show early tumor enhancement with washout. Corresponding peak enhancement map (E) and ^18^F-FDG PET/CT (F) shows higher peak enhancement of 0.33 mmol L^−1^ and lower tumor metabolic activity, SUV_max_ of 5.7, respectively.

Tumor imaging characteristics for all participants by pathological response are summarized in Table 2. In multivariable analyses, adjusted for pre-specified clinical variables: age, gender, tumor and nodal stage, peak enhancement was an independent predictor of response; odds of response decreased by 5% for each 0.01 unit increase (OR: 0.95; 95% CI: 0.90, 1.00; p = 0.04); AUC 0.87. There was no association between ^18^F-FDG PET variables and response (Table 3). Fig. 2, Fig. 3 provide illustrative examples of dynamic contrast-enhanced MRI and ^18^F-FDG PET/CT imaging for a responder and non-responder, respectively. Comparing pathological response to imaging response by RECIST v1.1 (taking Mandard TRG 1 or 2 and Complete Response or Partial Response by RECIST to both represent favorable response), n = 29/33 were concordant, n = 3/33 were discordant, and n = 1 became non-measurable due to stent insertion.

**Table 2:** Primary esophagogastric dynamic contrast-enhanced MRI and^18^F-FDG PET/CT characteristics according to pathological response (n = 33).

MRI	Responders (n = 11)		Non-responders (n = 22)		p-value^a^
	Median	IQR	Median	IQR	
Peak enhancement (mmol.L^−1^)	0.22	0.18, 0.34	0.35	0.25, 0.69	0.05
TTP (min)	0.78	0.59, 0.78	0.60	0.56, 0.78	0.77
iAUC	0.43	0.27, 0.65	0.26	0.16, 0.44	0.10
K^trans^ (min^−1^)	0.29	0.20, 0.37	0.20	0.13, 0.25	0.07
V_e_	0.31	0.20, 0.37	0.22	0.17, 0.33	0.65
k_ep_ (min^−1^)	0.83	0.53, 1.24	0.75	0.64, 0.90	0.45

^**18**^**F-FDG PET/CT**	**Median**	**IQR**	**Median**	**IQR**	
SUV_max_	10.0	5.4, 15.9	8.6	6.4, 13.1	1.00
SUV_mean_	5.5	3.2, 8.4	5.4	4.5, 7.6	0.95
Total lesion glycolysis	58.4	47.9, 91.4	56.3	23.0, 110.1	0.79
Metabolic tumor volume (cm^3^)	12.5	6.4, 15.4	9.0	6.2, 16.8	0.65

**Vascular-metabolic ratio**	**Median**	**IQR**	**Median**	**IQR**	
Peak enhancement: SUV_max_	0.027	0.018, 0.064	0.044	0.025, 0.073	0.29
K^trans^: SUV_max_	0.022	0.018, 0.044	0.020	0.015, 0.039	0.41

Abbreviations: time to peak (TTP), initial area under the receiver operating characteristic curve (iAUC), transfer constant (K^trans^), extravascular extracellular volume ratio (V_e_), rate constant (k_ep_), maximum standardized uptake value (SUV_max_), mean standardized uptake value (SUV_mean_), total lesion glycolysis (TLG), metabolic tumor volume (MTV).

a The reported p value is for Mann-Whitney *U* test.

**Table 3:** Multivariable analysis: assessment of imaging variables for prediction of response adjusted for baseline clinical information (age, gender, T stage, N stage) (n = 33).

	Odds ratio	95% Confidence interval	Area under ROC curve	p-value
MRI^a^				
Peak enhancement (mmol.L^−1^)	0.95	0.90, 1.00	0.87	0.04
TTP (min)	1.00	0.95, 1.05	0.74	0.92
iAUC	1.03	0.99, 1.07	0.80	0.15
K^trans^ (min^−1^)	1.13	1.00, 1.28	0.87	0.06
V_e_	1.01	0.97, 1.06	0.75	0.66
K_ep_ (min^−1^)	1.02	0.99, 1.05	0.78	0.30

^**18**^**F-FDG PET**				
SUV_max_	1.02	0.89, 1.18	0.76	0.76
SUV_mean_	1.03	0.79, 1.35	0.76	0.81
Total lesion glycolysis	1.00	1.00, 1.01	0.76	0.99
Metabolic tumor volume (cm^3^)	0.99	0.92, 1.05	0.77	0.67

Abbreviations: time to peak (TTP), initial area under the ROC curve (iAUC), transfer constant (K^trans^), extravascular extracellular volume ratio (V_e_), rate constant (k_ep_), maximum standardized uptake value (SUV_max_), mean standardized uptake value (SUV_mean_).

a MRI variable multiplied by 100 prior to analysis due to the small numerical values.

### 3.3 Associations with recurrence-free and overall survival

No relationships were identified between dynamic contrast-enhanced MRI or ^18^F-FDG PET/CT parameters and recurrence-free or overall survival (Supplemental Tables 3 and 4).

## 4 Discussion

There is an ongoing clinical need for better predictive markers to individualize care for patients with primary esophagogastric cancer as even with multimodality treatment, 75% of patients undergoing neoadjuvant therapy will be classed as pathological non-responders [16]. The tumor vascular-metabolic imaging phenotype may provide additional information to standard TNM staging.

To date there has been no published data of the vascular-metabolic phenotype in esophagogastric cancer. It has been proposed that a mismatch between vascularization and metabolism may occur as tumors enlarge, leading to localized hypoxia and anaerobic glycolysis [17]. Indeed, we noted no correlation between dynamic contrast-enhanced MRI and ^18^F-FDG PET variables in our cohort, where the majority were T3 cancers.

We also found that the initial tumor peak enhancement/SUV_max_ ratio was higher in patients who remained node positive after neoadjuvant therapy, suggesting possible predictive value for these parameters. Previous limited studies of the vascular-metabolic phenotype in colorectal cancer with perfusion CT and ^18^F-FDG PET have noted that tumors with a lower perfusion-metabolic ratio demonstrate higher VEGF and HIF-1 alpha expression [18]; and a combination of metabolic activity, permeability and perfusion may inform on outcome [19], but have not assessed response to neoadjuvant therapy.

In terms of the association of vascular-metabolic parameters with pathologic Mandard tumor regression grade, peak enhancement (representing the maximum gadolinium concentration prior to curve washout), alongside pre-specified clinical variables including gender, tumor and nodal stage, was a predictor of response. The odds of response decreased by 5% for each 0.01 unit increase in peak enhancement.

Our findings appear to differ from other studies of dynamic contrast-enhanced MRI in the response setting in esophageal cancer. However, of note, these studies have focused predominantly on squamous cell carcinomas treated with chemoradiotherapy, unlike our cohort, have not considered clinical factors, or used imaging metrics of response, which may account for the apparent difference in findings. For example, Lei et al. [20] (n = 25) and Sun et al. [21] (n = 59) identified squamous tumors demonstrating higher K^trans^ were more likely to undergo a favorable response to chemoradiotherapy, but this was defined by RECIST v1.1, not pathology. Similarly, Ye et al. (n = 237) found higher K^trans^ in responders treated with chemoradiotherapy [22]. Yet, in another cohort of squamous cancers (n = 32) treated with neoadjuvant chemoradiotherapy prior to surgery, Ji et al. [23] found no difference in baseline K^trans^ between complete (TRG 1 or 2) and incomplete responders. However, these were less advanced T1N0 and T2N0 stage tumors.

We found no apparent relationship between baseline ^18^F-FDG PET parameters and pathological response. Published ^18^F-FDG PET data are heterogeneous. For example, in a cohort of esophagogastric adenocarcinomas, Wieder et al. (n = 24) found no relationship between baseline SUV_max_ and response [24]. In contrast, Javeri et al. (n = 161) noted a lower initial SUV_max_ in non-responding adenocarcinomas treated with chemoradiotherapy [25]. In another cohort of squamous cancers undergoing definitive chemoradiotherapy Wang et al. (n = 138) found tumors with a high SUV_max_ (>11.9) had worse response, by RECIST [26].

In terms of recurrence-free or overall survival, while we found no apparent association between baseline vascular-metabolic parameters with oncological outcomes, several ^18^F-FDG PET studies have reported higher baseline SUV_max_ is associated with worse overall survival [[27], [28], [29], [30]], however, many others have not found any relationship [24,[31], [32], [33], [34], [35]].

Our findings are preliminary but suggest there may be predictive information in the extent of tumor enhancement beyond staging. Peak enhancement is a relatively straightforward parameter to assess in clinical practice without the need for pharmacokinetic modelling. Nevertheless, there are limitations to our study. Peak enhancement values may be affected by scanner and acquisition protocols; and physiologically, both tissue and circulatory properties contribute to its value [36]. Esophageal MRI is not without challenges, including the need to compensate for cardiac and respiratory motion as well as peristalsis. A major limitation is the small sample and low number of participants with events, particularly for multivariable analysis. To mitigate this, pre-specified clinical parameters were included given their known association with clinical outcome, and only a single imaging parameter assessed in this manner. We acknowledge that higher enrolment may have led to different conclusions and associations, not presently possible. Finally, this study included adenocarcinomas mainly, with only a few squamous cell carcinomas, but this reflects our practice.

In conclusion, in this exploratory study a high pre-treatment ratio of MRI peak enhancement to SUV maximum uptake was associated with persistence of nodal positivity following neoadjuvant therapy and surgery. MRI peak enhancement was also associated with pathological response suggesting potential additional information from functional imaging assessment.

## Credit author statement

Samuel J Withey: Data acquisition, Quality control, Data analysis & interpretation, Manuscript preparation. Kasia Owczarczyk: Data acquisition, Quality control, Data analysis & interpretation, Manuscript preparation. Mariusz T Grzeda: Data acquisition, Data analysis & interpretation, Statistical analysis, Manuscript preparation. Connie Yip: Study concepts, Study design, Manuscript editing, Manuscript review. Harriet Deere: Data acquisition, Manuscript editing, Manuscript review. Mike Green: Manuscript editing, Manuscript review. Andrew R Davies: Manuscript editing, Manuscript review. Gary J Cook: Study concepts, Study design, Data acquisition, Manuscript preparation, Manuscript editing, Manuscript review. Vicky Goh: Study concepts, Study design, Data acquisition, Quality control, Data analysis & interpretation, Manuscript preparation, Manuscript editing, Manuscript review.
