# Supplementary material for: Association of dynamic contrast-enhanced MRI and 18F-Fluorodeoxyglucose PET/CT parameters with neoadjuvant therapy response and survival in esophagogastric cancer
Source: Eur J Surg Oncol. 2023 Oct;49(10):None. doi: 10.1016/j.ejso.2023.05.009 (PMC10769883; doi:10.1016/j.ejso.2023.05.009)
Supplement: Multimedia component 1 [file mmc1.docx]

**Supplemental Material**

**Supplemental Table 1. 1.5T MRI acquisition parameters (MAGNETOM Aera, Siemens Healthcare).**

*Abbreviations: volume-interpolated breath-hold examination (VIBE); diffusion-weighted imaging (DWI); field of view (FOV); echo time (TE); repetition time (TR); number of signal averages (NSA); electrocardiogram (ECG)*

| **MRI Acquisition Parameters** | **T1 Dixon VIBE** | **T2 SPACE** | **DWI** | **T1 Dixon VIBE Post-Contrast** |
| --- | --- | --- | --- | --- |
| **Contrast** | - | - | - | 0.2 ml/kg Dotarem |
| **Plane** | Coronal | Axial | Axial | Axial |
| **2D/3D** | 3D | 3D | 2D | 3D |
| **FOV (mm)** | 365 x 449 | 337 x 450 | 343 x 440 | 349 x 429 |
| **Matrix** | 256 x 187 | 256 x 192 | 192 x 120 | 192 x 117 |
| **Voxel size** | 1.80 x 1.80 | 1.80 x 1.80 | 1.15 x 1.15 | 1.12 x 1.12 |
| **Slice thickness (mm)** | 1.8 | 1.5 | 6 | 5 |
| **Flip angle (degrees)** | 10 | 120 | 90 | 18 |
| **TE (ms)** | 2.4 | 158 | 61 | 1 |
| **TR (ms)** | 6.6 | 2665 | 1700 | 3.27 |
| **b-values** | - | - | 0, 400, 900 | - |
| **NSA** | 1 | 2 | 5 | 1 |
| **Gating** | Breath hold | ECG & Respiratory gated | Respiratory gated | Breath hold |
| **Temporal resolution (s)** | - | - | - | 6 |

**Supplemental Table 2. ^18^F-FDG PET/CT acquisition parameters (Discovery 710, GE Healthcare).**

| **PET/CT Acquisition Parameters** | |
| --- | --- |
| **CT tube voltage (kVp)** | 140 |
| **CT tube current (mA)** | Dose modulated (15-100) |
| **Reconstructed slice thickness (mm)** | 3 |
| **PET tracer** | ^18^F-FDG |
| **PET tracer activity (MBq)** | ~400 |
| **PET uptake time (min)** | 60 ± 5 |
| **PET detector** | Lutetium-based scintillator |
| **PET acquisition mode** | 3D |
| **PET scan time per bed position (min)** | 3 |
| **PET axial field of view (cm)** | 15.7 |
| **PET bed position overlap** | 11 slices |
| **PET coincidence window (ns)** | 4.9 |
| **PET energy range (keV)** | >425 |

**Supplemental Table 3. Univariate analysis: assessment of imaging variables for prediction of recurrence-free survival. Hazard ratio reflects 1 SD of change for each variable.**

*Abbreviations: positive enhancement integral (PEI), time-to-peak (TTP), initial area under the ROC curve (iAUC), transfer constant (K^trans^), extravascular extracellular volume ratio (V_e_), rate constant (k_ep_), maximum standardized uptake value (SUV_max_), mean standardized uptake value (SUV_mean_), total lesion glycolysis (TLG), metabolic tumor volume (MTV)*

|  | **Hazard Ratio** | **95% Confidence interval** | **p-value** |
| --- | --- | --- | --- |
| **MRI** |  |  |  |
| ***Qualitative*** |  |  |  |
| PEI (mmol.L^-1^) | 0.77 | 0.43 – 1.36 | 0.36 |
| TTP (s) | 1.03 | 0.59 – 1-79 | 0.92 |
| iAUC | 0.90 | 0.48 – 1.69 | 0.75 |
|  |  |  |  |
| ***Quantitative*** |  |  |  |
| K^trans^ (min^-1^) | 1.17 | 0.58 – 2.35 | 0.67 |
| V_e_ | 1.00 | 0.63 – 1.59 | 0.99 |
| k_ep_ (min^-1^) | 1.10 | 0.63 – 1.91 | 0.74 |
|  |  |  |  |
| **^18^F-FDG PET** |  |  |  |
| SUV_max_ | 0.69 | 0.35 – 1.37 | 0.29 |
| SUV_mean_ | 0.64 | 0.29 – 1.42 | 0.27 |
| TLG | 0.92 | 0.54 – 1.58 | 0.78 |
| MTV (cm^3^) | 0.95 | 0.60 – 1.50 | 0.82 |
|  |  |  |  |

**Supplemental Table 4. Univariate analysis: assessment of imaging variables for prediction of overall survival. Hazard ratio reflects 1 SD of change for each variable.**

*Abbreviations: positive enhancement integral (PEI), time-to-peak (TTP), initial area under the ROC curve (iAUC), transfer constant (K^trans^), extravascular extracellular volume ratio (V_e_), rate constant (k_ep_), maximum standardized uptake value (SUV_max_), mean standardized uptake value (SUV_mean_), total lesion glycolysis (TLG), metabolic tumor volume (MTV)*

|  | **Hazard Ratio** | **95% Confidence interval** | **p-value** |
| --- | --- | --- | --- |
| **MRI** |  |  |  |
| **Qualitative** |  |  |  |
| PEI (mmol.L^-1^) | 0.78 | 0.44 – 1.39 | 0.40 |
| TTP (s) | 1.12 | 0.59 – 2.14 | 0.73 |
| iAUC | 0.88 | 0.44 – 1.74 | 0.71 |
|  |  |  |  |
| **Quantitative** |  |  |  |
| K^trans^ (min^-1^) | 1.38 | 0.61 – 3.13 | 0.45 |
| V_e_ | 1.15 | 0.74 – 1.79 | 0.53 |
| k_ep_ (min^-1^) | 0.82 | 0.41 – 1.63 | 0.58 |
|  |  |  |  |
| **^18^F-FDG PET** |  |  |  |
| SUV_max_ | 0.80 | 0.38 – 1.70 | 0.56 |
| SUV_mean_ | 0.73 | 0.29 – 1.84 | 0.50 |
| TLG | 1.02 | 0.59 – 1.79 | 0.94 |
| MTV (cm^3^) | 1.00 | 0.62 – 1.63 | 1.00 |
|  |  |  |  |
